# A Case of Grade 2 Radiation‑Induced Osteosarcoma

**DOI:** 10.5334/jbsr.4175

**Published:** 2026-03-12

**Authors:** Flore Brachotte, Matthieu Wendling

**Affiliations:** 1 Medicine Resident in Radiology (5th year), CHU Liège Sart‑Tilman, Uliège, Belgium; 2 Radiologist, Department of Medical Imaging, Specialty in Osteoarticular Imaging, CHC Heusy Clinic, CHC MontLégia Clinic, Belgium

**Keywords:** radiation‑induced neoplasms, osteosarcoma, magnetic resonance imaging, positron emission tomography computed tomography, soft tissue neoplasms

## Abstract

*Teaching point:* The possibility of a radiation‑induced osteosarcoma should be considered in any painful, ossifying mass occurring within a previously irradiated area, even years after the initial treatment.

## Clinical Presentation

A 71‑year‑old man, previously treated in 2012 for a dedifferentiated liposarcoma of the left thigh with surgery and radiotherapy, presented with progressively worsening and disabling pain over several months, localized to the proximal third of the previously irradiated thigh.

## Imaging Findings

**Figure 1:**
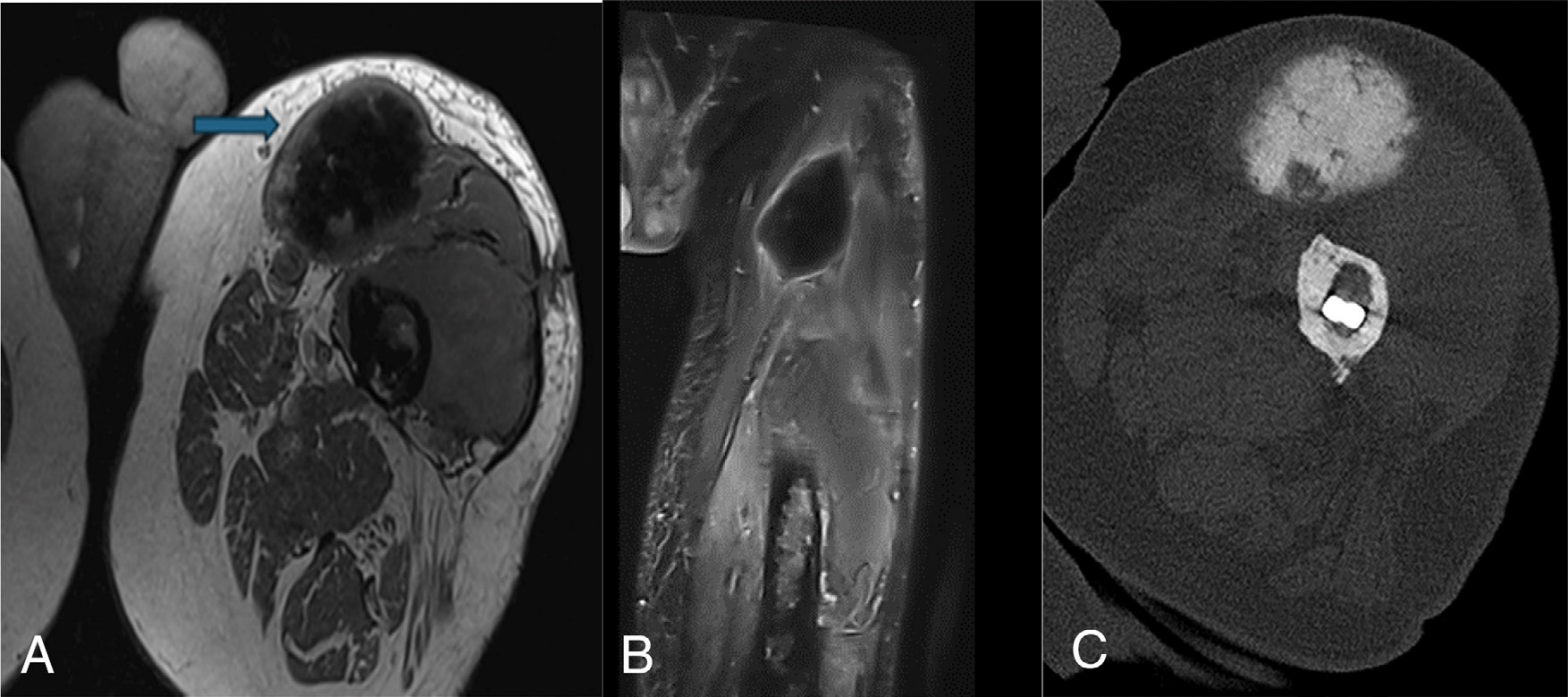
MRI. T2 DIXON **(A)** and post‑contrast T1 DIXON **(B)** sequences following intravenous gadolinium administration demonstrate a hypointense T1/T2 mass within the sartorius muscle with peripheral enhancement. **(C)** CT scan without injection in bone window. Individualization of an ossifying mass.

Plain radiographs revealed a densely radio‑opaque mass. MRI demonstrated a large intramuscular lesion measuring 66 mm within the sartorius muscle, appearing hypointense on both T1‑ and T2‑weighted sequences, with irregular margins (Figure 1A), and showing peripheral enhancement after gadolinium injection (Figure 1B). Myositis ossificans was initially considered as a differential diagnosis due to the ossifying nature (Figure 1C).

18F‑FDG PET‑CT revealed partial peripheral hypermetabolism with a suspicious appearance. A percutaneous biopsy yielded limited cellularity but revealed cellular atypia within an ossifying stroma.

Given the history of prior irradiation, progressive radiologic changes, and partial histologic features, the diagnosis of a radiation‑induced sarcoma (RIS) was retained. The patient received three cycles of chemotherapy, followed by surgical resection. Histopathological analysis of the surgical specimen confirmed a grade 2 osteosarcoma (FNCLCC).

**Figure 2:**
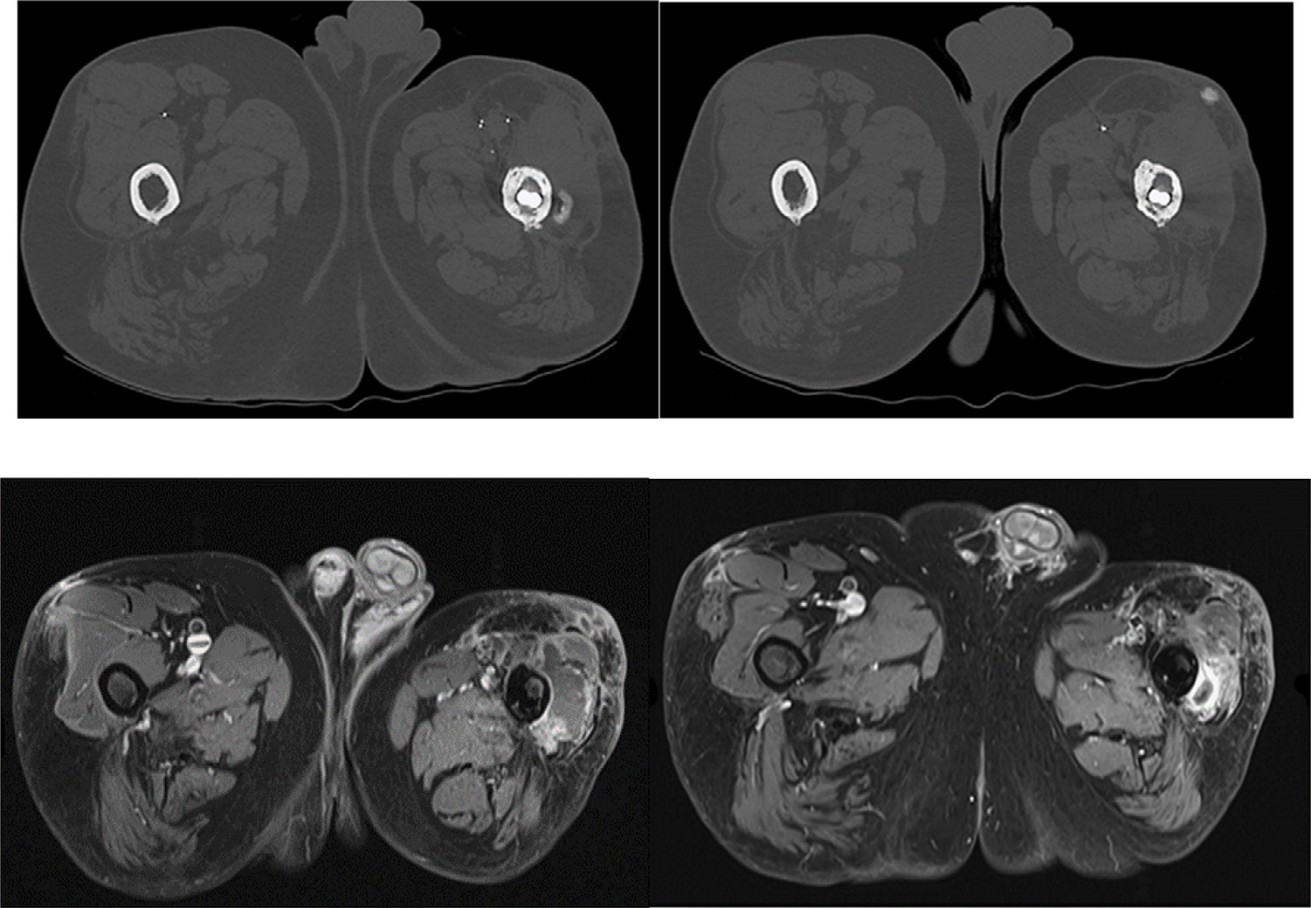
CT scan with bone window and MRI T1‑weighted fat‑saturated sequences after intravenous injection of gadolinium complex.

A follow‑up MRI at three months revealed two similar recurrent nodular lesions, measuring 20 mm adjacent to the femoral diaphysis and 11 mm in the anterior subcutaneous tissue, both displaying hypermetabolism on PET‑CT (Figure 2).

## Final Diagnosis

Grade 2 radiation‑induced osteosarcoma.

## Commentary

RIS represent less than 1% of post‑radiotherapy complications. The latency period often exceeds 10 years. Radiation‑induced osteosarcoma is a rare entity with a poor prognosis due to its aggressive behavior [1]. Diagnostic criteria for RIS include a prior history of radiotherapy (usually >3 years), tumor development within the irradiated field, and histological distinction from the primary tumor.

Imaging plays a critical role in identifying abnormal masses within irradiated areas. When suspicion arises, PET‑CT combined with targeted biopsy is essential to confirm malignancy. Management typically involves wide surgical excision, often combined with chemotherapy.
